# Research highlights

**DOI:** 10.1111/eva.12146

**Published:** 2014-01-27

**Authors:** Britt Koskella

**Affiliations:** Evolutionary Applications

With a changing climate, increasing need for food security, growing threats to marine and freshwater fisheries, an impending antibiotic crisis, invading species, and continued risks of emerging human and agricultural diseases, the need for creative and forward-thinking solutions is critical. The application of evolutionary theory to these everyday problems is proving to be a reliable and powerful tool, without which our ability to predict the responses of populations to change, both human-mediated and otherwise, is not possible.

Evolutionary Applications is the only journal specializing specifically in publishing papers that make contributions to fundamental questions in evolutionary biology using study systems that are of practical or applied importance in topics including, but not limited to: agriculture, aquaculture, biomedicine, biotechnology, climate change, conservation biology, disease biology, forestry, invasion biology, and fisheries and wildlife management. As such, one of our main goals is to promote interest of applied evolution to a diverse audience including evolutionary biologists, ecologists, biomedical researchers, environmental consultants, and biologists within industry, government, and health care. Toward this end, this new series of research highlights will offer brief synopses of new work with direct relevance to readers of Evolutionary Applications from across other journals with the aim of exploring the breadth of potential applications of evolutionary theory from across fields and disciplines.

This past year was full of noteworthy evolutionary applications, including a strong focus on the use of combination therapy to slow or stop the spread of drug resistance. In particular, the application of evolutionary theory to resistance of cancers was a hot issue, with a special issue of *Evolutionary Applications* (volume 6, issue 1), a *Nature Reviews Cancer* piece about the importance of life-history trade-offs to our understanding of cancer progression (Aktipis et al. 2013), a *Nature* review article on genomic instability of cancer (Burrell et al. 2013), a Jacques Monod conference on ‘Ecological and evolutionary perspectives in cancer,’ and the 2nd International Biannual Evolution and Cancer Conference. This blossoming interest is uncovering the roles of evolutionary processes in shaping the development, spread, and virulence of cancers, but is also highlighting the current gap in our ability to translate evolutionary theory into successful treatment. An elegant example of the great potential of this approach is the recent work by Shi and coauthors identifying two core resistance pathways in metastatic melanomas (Shi et al. 2014). The researchers found that drug resistance of these cancers occurred most often via two distinct ‘drug escape pathways,’ explaining 70% and 22% of resistance among disease-progressive tissues. These data act as strong justification for the use of combination treatment to target both BRAF inhibitor resistance pathways simultaneously to block these common routes of drug escape.

Similar approaches are being applied to tackle the current antibiotic crisis, as has been reviewed in a recent *Nature Reviews Genetics* article (Palmer and Kishony 2013). The authors point out the importance of using optimal combinations of antibiotics, ideally chosen based on negative cross-resistance and antagonistic interactions among resistance mechanisms. However, a recent paper by Pena-Miller and colleagues, combining experimental and genomic techniques with mathematical modeling, highlights that caution must be employed when employing combination treatments, as the use of synergistic antibiotics can in fact increase pathogen load under suboptimal treatment durations or subinhibitory drug concentrations (Pena-Miller et al. 2013). A similar warning has now been raised for combination treatment of malaria due to the discovery of high association among resistance alleles in multiple regions of China (Ding et al. 2013). Furthermore, a clear understanding of epistasis among resistance mutations is key to predicting the spread of multidrug-resistant bacteria, as has been nicely demonstrated by Borrell et al. (2013). They found that combinations of drug resistance mutations in *Mycobacterium smegmatis* showing a competitive fitness advantage *in vitro* were also the most frequently observed combinations found in multidrug-resistant clinical isolates of human tuberculosis in South Africa.

The application of evolutionary theory to development of combined treatments is certainly not limited to human disease. Experimental evolution of the green chlorophyte, *Chlamydomonas reinhardtii*, was used by Lagator and coauthors to demonstrate the utility of combined herbicides for slowing the evolution of resistance in agricultural settings (Lagator et al. 2013). However, their data come with a similar word of warning: using these combinations at low doses can select for both increased rates of resistance and a more general resistance mechanism relative to single herbicide treatment.

This small subsample of recent research published in Evolutionary Applications and other high-profile journals on combination treatment emphasizes the power of applying evolutionary theory to treatment design, both in terms of creating more ‘evolution-proof’ combinations and in predicting potential negative consequences of this approach. We look forward to future research on the topic and continued monitoring of treatments designed in the light of evolutionary theory.

## References

[b1] Aktipis CA, Boddy AM, Gatenby RA, Brown JS, Maley CC (2013). Life history trade-offs in cancer evolution. Nature Reviews Cancer.

[b2] Borrell S, Teo Y, Giardina F, Streicher EM, Klopper M, Feldmann J, Gagneux S (2013). Epistasis between antibiotic resistance mutations drives the evolution of extensively drug-resistant tuberculosis. Evolution, Medicine, and Public Health.

[b3] Burrell RA, McGranahan N, Bartek J, Swanton C (2013). The causes and consequences of genetic heterogeneity in cancer evolution. Nature.

[b4] Ding S, Ye R, Zhang D, Sun X, Zhou H, McCutchan TF, Pan W (2013). Anti-folate combination therapies and their effect on the development of drug resistance in *Plasmodium vivax*. Scientific Reports.

[b5] Lagator M, Vogwill T, Mead A, Colegrave N, Neve P (2013). Herbicide mixtures at high doses slow the evolution of resistance in experimentally evolving populations of Chlamydomonas reinhardtii. New Phytologist.

[b6] Palmer AC, Kishony R (2013). Understanding, predicting and manipulating the genotypic evolution of antibiotic resistance. Nature Reviews Genetics.

[b7] Pena-Miller R, Laehnemann D, Jansen G, Fuentes-Hernandez A, Rosenstiel P, Schulenburg H, Beardmore R (2013). When the most potent combination of antibiotics selects for the greatest bacterial load: the smile-frown transition. PLoS biology.

[b8] Shi H, Hugo W, Kong X, Hong A, Koya RC, Moriceau G, Lo RS (2014). Acquired resistance and clonal evolution in melanoma during BRAF inhibitor therapy. Cancer Discovery.

